# Game on: Can gamification enhance productivity?

**DOI:** 10.12688/f1000research.131579.1

**Published:** 2023-07-12

**Authors:** Habeeb Ur Rahiman, Rashmi Kodikal, Sucharitha Suresh

**Affiliations:** 1 College of Business Administration, Kingdom University, Manama, Manama, Bahrain; 2 Department of Management, Graphic Era (Deemed to be University), Dehradun, Uttarakhand, India; 3 Department of Hospital Administration (Allied Health Science), Father Muller Medical College, Mangalore, Mangalore, India

**Keywords:** Gamification, motivation, adoption, usefulness, productivity, Job Engagement

## Abstract

**Background:** Research suggests that gamification can increase work engagement by providing employees with a sense of autonomy, competence, and relatedness, and by creating a fun and engaging work environment. Gamification is designed to increase consumer and employee engagement and see that they holistically collaborate to achieve a shared vision. The concept of gamification is as old as learning itself, just that the use of the terminology “Gamification” is of a recent origin.

**Methods:** This article focuses on the impact of gamification in various organizations and simultaneously sees its relationship with job engagement and productivity. A primary investigation was done to determine the nexus between the various variables and data collection from 400 respondents working in various fraternities of the economy from both public and private domains from countries in the Gulf region. The structural equation model and SPSS has been inferred to analyse the results.

**Results:** The study results show that variable such as perceived adoption and usefulness in the gamified system is significantly associated with job engagement. Similarly, employee’s recognition and perceived motivation have a positive impact on productivity. The study identified job engagement mediating factor to enhance organisational productivity in a gamified system.

**Conclusion:** The effectiveness of gamification in enhancing work engagement may depend on factors such as the design of the gamification system, the preferences and motivations of individual employees, and the organizational culture and goals. The findings have significant implications for insight into how employees in the service sector are aware of the gamified working environment and react to the system through work engagement and productivity.

## Introduction

The precipitously changing workforce dynamics paired with digitization and switching work preferences compel the constant evolvement of practices and procedures ( Ashley et al., 2022). In an era of high-speed digitalized technology and a multi-generational capacity team, the scenario has resulted in a struggle for the smarted of the people ( Palmquist, 2020). The critical need for firms to entice talent becomes a fascinating case to embrace innovative operational and recruitment methods, such as gamification ( Morschheuser et al., 2018). Gamification can increase employee engagement by incorporating game-like elements into non-game contexts such as work, to create a more interactive and engaging experience ( Kapp, 2012). By using features such as points, badges, and leader boards, gamification can provide employees with a sense of accomplishment, feedback, and recognition for their work, which can increase their motivation and engagement. The concept of gamification is as old as learning itself, just that the use of the terminology “Gamification” is of a recent origin ( Prince, 2013). To make learning and work interesting, use of non-computer-aided techniques has been seen for centuries. However, in the early 1970s and 80s with video games becoming popular, the gamification concept got coined a new label. The term gamification is the most trending and widely applied concept in a non-game context ( Buckley et al., 2019). Globally, approximately 40% of the fortune 1000 companies successfully applied gamification in the workplace ( Keepers et al., 2022). This progress makes gamification one of the highly important innovative developments in the administration of organizations ( Grünewald et al., 2019; Thompson & Irvine, 2014). Technical progress has led to numerous alterations in areas of human resources management (HRM) to connect with the needs of globalization ( Woodcock & Johnson, 2018). The acceleration of artificial intelligence, the internet of thinking and machine learning, And other advancements in the field of technology. have bought revolutionary changes in industrial routine operations ( Dessureault, 2019; Jia et al., 2017). This digital enhancement affects various stages of organizations, and it needs to adapt to advancements as new methods of working. Human resource (HR) and people management in organizations demand this technology adoption in various modes which has influenced HR operations ( Medeiros et al., 2015). In recent years, several methods have been created to encourage and help the workforce accomplish desired behaviors. Since a human being has an instinctive pleasure of playing, progress in this space that is gradually obtaining consideration is gamification ( Warmelink et al., 2020).

Gamification is applied in several organizational disciplines from manufacturing, operations, and recruitment, staff development activities ( Deif, 2019). Artificial intelligence, machine learning, and the internet of thinking are mostly adopted by organizational routine operations. There is a strong correlation between artificial intelligence (AI), machine learning (ML), and internet of things (IoT) with gamification with various tools which helps to enhance better performance ( Bahadoran et al., 2023; Jacob et al., 2022). Currently, companies are developing frameworks and tools considering AI technology as a form of the game to identify candidate skills and also utilize gamification to avoid recruitment bias. Human resource specialists had reacted to global digitalized transformation all through advanced platform methods known as gamification ( Ramallo-González et al., 2022). Gamification can be started by shifting organization strategy and facilitating the organization in several areas such as operation, recruiting training, and development. Notable organizations like European Central Bank ( Donovan, 2011), SAP ( Kotsis et al., 2021), Samsung ( West & Lockley, 2016), and Apple ( Favorskaya et al., 2015), have applied gamification in their organizational activities and practices. In the human resource system, gamification comprises incorporating gaming elements and motivating techniques for example through leader boards, and points, into HR practices to design routine tasks and procedures that are identified by operators as game-like practices ( Scurati et al., 2020). In addition to companies promoting the enactment of gamification for their workplaces to improve efficiency, productivity, and enthusiasm, some companies also use it for pre-emptive purposes, such as a vendor or competitive analysis) ( Schlömmer et al., 2021). The outcomes of gamification in companies eventually depend on whether the workforce is encouraged to apply it, and whether gamification improves their optimistic opinions related to their employment ( Metwally et al., 2021). If the workforce feels more pride, gratification, and engagement in their task as a cause of gamification, that will reflect in organizational productivity ( Guven & Sakamoto, 2016; Kaur et al., 2021). With the growth of the E-Human resource management system, the digital revolution has transformed the traditional business process by applying game-thinking in the organizational work process ( Behl, Sampat, et al., 2021). To meet organizational goals, one needs to have strong gamification functions that will be utilized during the employment practice and create an advantage for the company and workforce ( Zhang et al., 2021).

Our research instrument was used to understand how industrialists and professionals in the Gulf cooperation council (GCC) perceive the concept and practice of gamification. Therefore, our research was taken up to examine the impact of gamification in various organizations and simultaneously see its relationship with job engagement and productivity. The primary objective of this research is to identify the role of perceived usefulness, motivation, adoption, and recognition in the organization while adopting gamified tasks in an HR system.

In the first section of the manuscript, the paper reviewed flow theory and gamification, and the components and mechanisms of gamification that exist in this domain. The methodology has been explained in the second section, followed by results, and a discussion was presented. The paper ends with a conclusion and suitable theoretical implications and direction for limitations and future research.

### Background

Numerous studies have been conducted by researchers globally have discussed that the digital revolution appears to have a significant relationship between efficiency, motivation, work engagement, and productivity. From the review of previously published literature, we can infer the impact gamification will have on organizational operations in various areas.

### Flow theory and gamification

Gamification is a mechanism or element of the game to make available affordance for game events in non-game environments ( Brandstätter & Sommerer, 2016; Müller et al., 2016). It is difficult to claim when the idea of gamification surfaced. Several individuals opined that gamification drew its origins in the 20 ^th^ century when the boy scouts organization was established ( Gatautis et al., 2021). Since then, gamification has remained trail blazing in web-related fields and elsewhere. Organizations use gamification to maintain workforce engagement ( Prasad et al., 2019), incentivize users ( Naeem et al., 2017), recruit, lead, and enhance productivity ( Silic & Back, 2017). Flow theory suggests that individuals experience a state of optimal experience and engagement when they are fully immersed and focused on an activity that is challenging but within their skill level. Gamification can be designed to enhance the experience of flow by providing users with clear goals, immediate feedback, and a sense of progress and accomplishment ( Hammedi et al., 2021; Murray, 2018). Gamification has in recent times been offered as a favorable prospect to enhance human resource management (HRM) systems and instruments. The purpose of game design components, such as badges ( Lee et al., 2016), leader boards, and points have become a recognized exercise all over society ( Donnermann et al., 2021). Integration of corporate environmental responsibility and pro-environmental action into the gamified system or game design is an essential element of the gamification approach ( Morganti et al., 2017). To enhance environmental performance, it is important to encourage pro-environmental and pro-social behaviour ( Marculescu et al., 2020). Therefore, incorporating pro-social aspects in the gamification process by focusing on the benefits of the elements of the gamified system can increase the likelihood of user engagement in pro-environmental and social aspects. Over the years, an immense quantity of research has been conducted to create structures and categorizations for gamification and game model components ( Patrício et al., 2018; Zhang et al., 2021). Researchers have discovered methods, layouts, design, styles, and, most recently, the effects of gamified organizations ( Beard-Gunter et al., 2019; Patrício et al., 2018) in order to popularise the concept of gamification in the field of HRM. Of recently, the research on gamification focuses on involving methods to examine potential consequences of gamifying the HRM system and tools ( Makanawala et al., 2013).

### Components and mechanism of gamification

The engaged workforce tends to be energetic, passionate, and determined to stage their roles. New methods can enable such engagement by establishing resource exchanges, communications, and mutual well-being ( Silic et al., 2020). However, such engagement necessitates careful supervision ( Hammedi et al., 2021). Globally notable companies have implemented and experienced designing fun events as a method for boosting workforce engagement, with the perception that fun can improve employee’s work satisfaction and commitment and eventually enhance their well-being and productivity ( Gatautis et al., 2021).

Several studies believe that an effective work environment is the major indicator of workforce well-being, However, such a reflexive form of well-being can be accompanied by more effective methods, for example, job engagement ( Gerdenitsch et al., 2020). Well-engaged employees have optimistic appraisals of their employment condition, and beyond mere gratification, they are encouraged to disburse strength to achieve a task; they also recognize their inspiration ( Rapp, 2020). Therefore, both work environment and job engagement appear vital for the well-being of the workforce ( Behl, Sheorey, et al., 2021; Patrício et al., 2018). Advanced creative technologies, as well as the attractiveness of digital games, approach a unique opportunity for generating a fun or positive environment in the workplace ( Pierce, 2019). That is, executives can take advantage of game-based project standards and accept the configuration, appearance, and sense of a game to get employee’s skills more constructive, pleasant, and exciting for members of the organisation, which may well improve managerial objectives ( Bouzidi et al., 2019; Browne et al., 2018; Sanmorino et al., 2021).

In gamification, the entertaining and engaging environments usually observed in games are implemented to improve employees’ dedication to and engagement in real-life creative activities ( Mitchell et al., 2020). For example, to enhance the effective learning process, the training and development unit implements certain gaming mechanisms and components to gain potential results ( Leon & Peña, 2022). Team building, communication, logical reasoning, situation analysis, body language theory, and brainstorming activities. are the major components of gamification in a certain organisation ( Silic et al., 2020). Companies utilise gamification in many aspects of the operation aligning with AI to enhance job engagement. Moreover, AI, ML, and IoT provide more opportunities for employees to concentrate on core issues and provide automation in many operational processes ( Marculescu et al., 2020). The combination of gamification with AI, IoT, and ML could create an improved outcome in various tasks. This combination for example in recruitment, many companies use AI and gamification tools to shortlist candidates. Companies may use tools like ‘Scoutible’ a short game developed to determine the ability of the candidates to a particular task. Similarly, tools like Kahoot and Mentimeter are used to improve engagement and enhance interpersonal skills ( Moorhouse & Kohnke, 2020). AL or ML combined games can also predict candidates’ thoughts, personalities, and decision-making capabilities by mapping the metrics required for a particular task and identifying the overlapping skills and qualities ( Kranthi Kumar et al., 2020; Marculescu et al., 2020).

The potential use and benefits of the gamified system are described by flow theory. This theory proposes that an individual could achieve a status of flow, distinguished by the comprehensive concentration in the situation when completely engaged in executing a task ( Moneta & Csikszentmihalyi, 1996). The concept of flow can be felt in several distinct situations. Artists can achieve flow while acting as a character, and athletes might feel it while playing at the boundaries of their physical ability ( Patrício et al., 2018). Likewise, workers can achieve flow by feeling concentration or inclusion, enthusiasm, and enjoyment of the assignment they execute ( Duggal & Gupta, 2020). Thus, we postulated that the beginning of gamification in the human resource management approach may influence employee engagement and productivity through perceived adoption, recognition, usefulness, and motivation ( Brangier & Marache-Francisco, 2020; Whitson, 2013).

### Development of hypothesis

**Figure 1.**
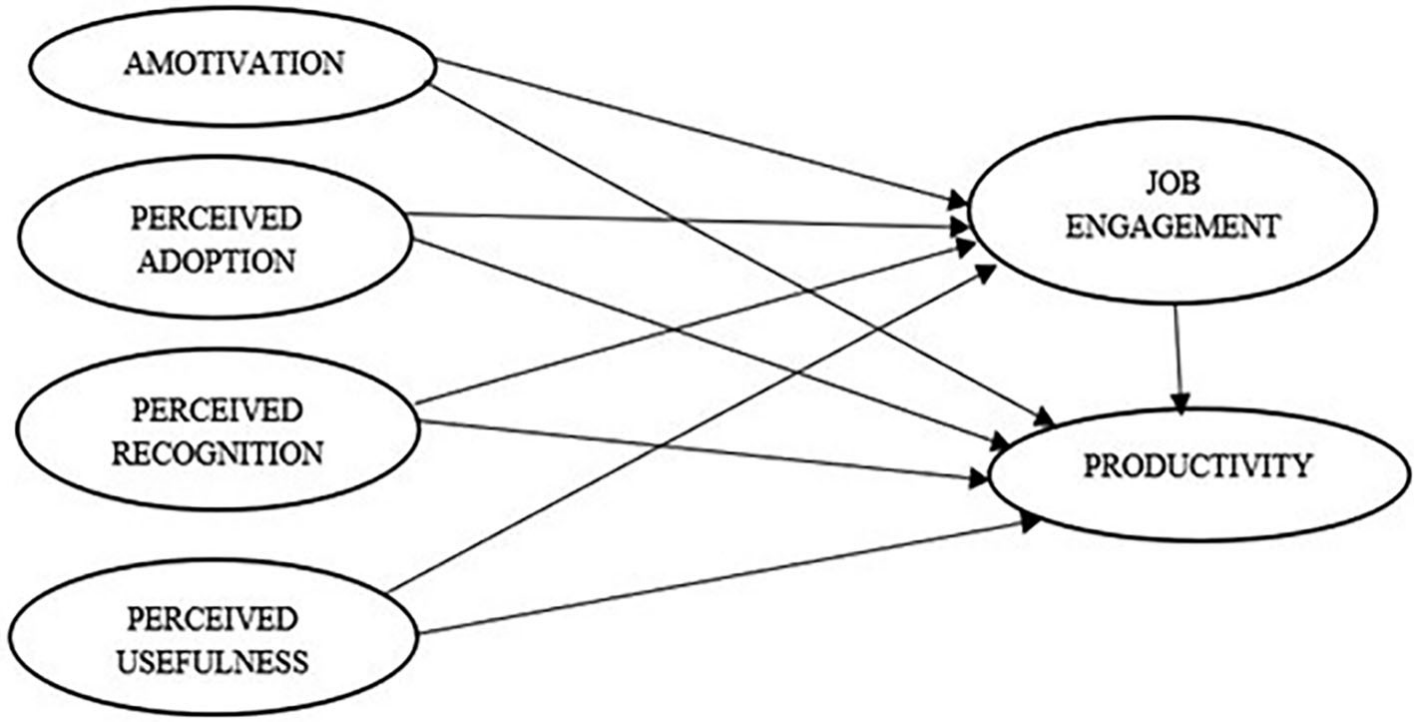
Proposed conceptual model. Source: Authors’ development.

Our research proposes that gamified workplace practice could enhance job engagement and productivity of employees through four factors namely perceived adoption, recognition, usefulness, and motivation ( Figure 1). In this research, we examined how these aspects are related to better job engagement and productivity at the workplace and how they help in establishing potential mediating methods through which they correlate to the results. The entire paper was vested around testing the interrelationship between these variables and they are pictorially represented as follows:

Job engagement has been defined as an ‘individual’s enthusiasm and involvement in a task assigned to them ( Metwally et al., 2021). The highly involved individuals are usually motivated since they are identified in their jobs ( de la Peña et al., 2021). They tend to work more efficiently and productively ( Behl, Jayawardena et al., 2021). Adoption of a gamified task in an employee’s role and applying those features to involve more efficiently in their organizational task ( Moneta & Csikszentmihalyi, 1996) will likely transform the work environment into a productive one, which supports the interconnection between experiencing the game and appreciating work. Therefore, we affirm that if an individual appreciates the gamified HRM or operational method and realizes it is enjoyable, thrilling, or fascinating, applying gamification quickly to their organizational task is likely to improve job engagement. Therefore, Hypothesis 1, states as follows:

*H1: Employees’ perceived adoption in the gamified HRM system is significantly associated with job engagement.*

Recognition indicates the opinion collected from the society, which causes by kinds of employee engagement that can take up the type of online engagement or accomplishment ( Murray, 2018). A well-structured recognition system helps organizations to enhance productivity ( Kour et al., 2019). Recognition can be merely explained as the explicit opinion collected through an HRM system on job-related activities ( Jeske et al., 2021), and it is designed through the actions that employees examine ( Zitars et al., 2021). Recognition often establishes reciprocal behavior whereby a worker could either obtain or offer feedback ( Magni et al., 2021), which results in generating more respect and advantages for the whole HRM system as additional relationships and community interactions are made ( Ángeles López-Cabarcos et al., 2021). Further gaining positive recognition from colleagues or line managers encourages a worker’s enthusiasm to identify others mutually and reciprocally while utilizing a service, indicating that obtaining recognition generates productivity ( Sadick et al., 2020). The performance of employees is often associated with effective performance appraisal and reward systems ( Adin, 2021). Gamification or gamified HRM systems can positively create an automated recognition environment that results in productive workforce Considering the importance of recognition in the human resource system, Hypothesis 2 is proposed.

*H2: Employee recognition in the gamified HRM system has a significant impact on productivity.*

Employees’ perceived usefulness of gaming signifies the degree of confidence in job engagement after applying a gamified HRM system ( Küpper et al., 2021). Job engagement is associated with developing the performance and efficiency in job-related responsibilities ( Aubert & Lienert, 2019). Prior findings recognized a relationship between usefulness and job engagement ( Golrang & Safari, 2021). If workers realize that the gamified technique is beneficial to them in their job, it will be possible to also realize their engagement in tasks and productivity to increase as a consequence of the executed approach ( Sanchez et al., 2020). In a gamified system, individual engagement is through internal communication, familiarity with tasks, and enthusiasm for their assignment ( Höllig et al., 2020). For instance, the gamified HRM approach could enable more efficient communication over the shared interaction among the workforce, which relates to the development of the game features ( Mullins & Cronan, 2021). Therefore, due to advanced interaction and usefulness, it is feasible for the workforce to perform their job more effectively and ambitiously, thus developing engagement in the organizational tasks ( Diefenbach & Müssig, 2019). From an organizational view, we are further likely to see a significant influence on job engagement if the organisational system with the gamified mode is employee-friendly to use and beneficial in performing tasks ( Treiblmaier & Putz, 2020). Hence, if gamification is helping the workforce to improve their performance and efficiency in outcomes, it must have the advantage to improve their efficacy and overall enthusiasm. Subsequently, perceived usefulness must be positively impacted by an employee’s job engagement. Accordingly, hypothesis 3 can be proposed as follows.

*H3: Employees perceived usefulness in the gamified system is positively associated with job engagement.*

Motivation is a vital factor for an individual to convert their energy into a productive outcome ( Jeha et al., 2022). Motivation encourages workforce commitment and engagement in tasks more effectively than unsatisfied or demotivated manpower ( Albro & McElfresh, 2021; Heyns et al., 2021; Owen et al., 2018; Stirpe et al., 2021). Motivation is the intensity of a person’s motivational feel while engaged in an occupation. Prior findings have proved a positive association between motivation and productivity through job engagement ( Tziner & Tanami, 2013). Individuals’ self-motivation often leads to better efficiency and performance in tasks ( Saks, 2021). Therefore, we affirm that when workers are well interested to apply the gamified HRM and operational approach, it provides greater amounts of job engagement and productivity due to the significant impacts of the motivational aspects that enhanced the employee’s well-being and emotional association with the organization. Gamification can be a useful tool for motivating employees inside an organisation, and motivating employees is positively correlated with productivity, a crucial result that companies are interested in. Therefore, hypotheses 4 and 5 are proposed in the following way.

*H4: Employees perceived motivation in the gamified system is significantly associated with productivity.*

*H5: Employees’ Job engagement is mediating factor to enhance productivity.*

## Methods

### Ethical considerations

The adopted questionnaire was submitted to the ‘Academic Integrity and Ethics Committee’ of the College of Business Administration, Kingdom University, and got approved as per the research policy and procedure on 5 ^th^ June 2022 with ref. no. (CBA/30/22). The data collected will be used only for academic research purposes. All the respondents who participated in this survey have given their written informed consent to participate in the study and use their feedback to publish in our research publications. The consent of the participants has been asked at the commencement of the questionnaire and the participants responded by accepting the statement in the instrument link “I am willing to participate in this survey”. After expressing their consent, rest of the questionnaire appears for the respondents and data has been considered for analysis. To protect the participants’ interest, personal information is kept confidential.

### Research design

The current quantitative research explains the casual relation between role of gamification towards productivity and job engagement. The study was conducted from June 2022 to first weeks of February 2023 in Gulf Cooperation Council countries. The entire research framework was based on the primary investigation, and an online survey method was utilized to achieve the objectives of the research work. Data was chosen from various pools of countries located in the gulf region, and the service sector was predominantly chosen for sampling. The multistage sampling method was used for the choice of respondents from public and private domains. The respondents were rendering services in IT, banking, education, and the telecom sector. These respondents were chosen because of their proximity to work with information technology and with gadgets where gamification could be easily made accessible. The countries chosen for sampling were UAE, Saudi Arabia, Oman, Bahrain, Kuwait, and Qatar. Proportional samples have been drawn from countries in the Gulf region. The researcher’s familiarity with regions and their access to organizations enabled a judgment-based sampling. As judgment-based sampling was used to exert discretion in the choice of respondents, only those companies that used gamification concepts in the workplace participated as respondents. Meanwhile the missing data were addressed by deleting the incomplete cases.

### Samples and selection criteria

The respondents were chosen from the organizations that adopted the gamification approach in their operations in the Gulf region. The researcher approached the human resource department of the selected companies both private and government ownership and distributed a questionnaire (online link) to respondents in various departments. A total of 600 respondents from all these countries were invited to participate in the survey from June to December 2022, and the researcher managed to receive 400 complete responses based on judgemental sampling methods. The number of samples was determined based on ( Burmeister & Aitken, 2012), considering the number of organizations that adopted gamification in their routine operations.

The selection of participants initiated the administering cluster approach since samples were collected from different parts of the gulf countries. To avoid bias in sample selection, we used a multi-stage sampling approach, so that can ensure that the sample is more representative of the population by including a range of different groups or clusters.

### Data analysis and interpretation

The data has been analyzed applying SPSS (version 26) and identified descriptive statistics to summarize the characteristics of a quantitative variable, such as the mean, median, standard deviation, and range. Similarly, inferential statistics such as t-tests, ANOVA, regression analysis, and correlation analysis, are used to test hypotheses or make predictions about a population based on a sample of data. Similarly, SPSS Amos version 26 software is used to test complex relationships among variables. The open access alternative for SPSS Amos is Microsoft Excel. Amos also testified the model against the observed data and estimated the model parameters (factor loadings, regression coefficients, and error terms), examined the goodness-of-fit statistics (chi-square, RMSEA, CFI) to determine if the model fits the data.

### Instrument and measurement of variables

The research frame comprised a sample set of 400 respondents drawn based on the judgemental sampling method. The questionnaire was distributed to 600 people out of which only 400 responses could be utilized due to faulty filling or lack of response. After removing the invalid responses, statistical analysis was conducted and a pilot study was conducted before reaching out to them. The sample size of 400 demonstrated sufficient statistical control. A 5-point Likert scale has been utilized to understand the opinion of the respondents regarding the variables. The instrument was predominantly adopted from the past literature and each construct was determined with three or four items using a Likert scale in the design ranging from one to five. The research instrument was categorized into four parts: Section 1: Demographic information (six items); Section 2: Gamification questions (three items) adopted from ( Seaborn & Fels, 2015); Section 3: Details about perceived adoption (for example, application of gamification will accomplish tasks, improve my performance, enhance my effectiveness, make my job easy, four items) adopted from ( Hu et al., 2023); Section 4: Details of perceived recognition (for example, colleagues must recognize my performance in newly adopted jobs, considering my feedback in new system *etc.*, four items) adopted from ( Ortiz-Rojas et al., 2019); Section 5: Details of Perceived Usefulness (For example: Using gamified system improves performance, increases productivity, enhances effectiveness in job *etc.,* four items) adopted from ( York & deHaan, 2018); Section 6: Motivation (For example; classified into intrinsic motivation, identified regulation, external regulation, amotivation, four items) adopted from ( Zimmerling et al., 2019); Section 7: Productivity (for example; developed team spirit and believe in achieving business goals; ability to market product; contributing profit generation; ability to make quick and qualitative business decision, four items) adopted from ( Zainuddin et al., 2020); Section 8: Job engagement (for example; exerting full effort into my job; hardest to perform well on job; absorbed by my task job, concentrated on my task *etc.*, eight items) adopted from ( Patricio et al., 2022).

The details of the results, SEM, and the statistical relevance of the tests conducted are summarised in the next few sections.

## Results and findings

### Demographic details and the relationship of independent variables on the demographic constructs

A glimpse of the descriptive variables in terms of demographic aspects and usage of gamification is listed below in Table 1. As depicted in Table 1, the target respondents mainly comprised of women (60.5%) in the age group of 31 to 40 years old which accounted for the majority of the population. In terms of their educational qualification, 49% had acquired a graduation degree (bachelor’s and post bachelor’s degree) or higher than the graduation degree. It was noted that 61.5% who responded were rendering services in the private sector and were working in the position of a team member and not in a managerial position. The survey-based study also included questions about their opinion about gamification and its usage in their daily life. 82% of the respondents were aware of gamification and 74% of them used gamification in their normal life. But only 9.5% of the respondents used it daily and 14.5% were occasional users and the rest of them hardly used gamification for their daily entertainment. Hence it can be inferred that most of the respondents did not depend upon gamification or any gamification-related mechanism for perceived enjoyment or interactions.

**Table 1.** Demographic details.

		N	% of sample
Gender	Men	158	39.5%
	Women	242	60.5%
	Total	400	100.0%
Age	Below 20 years	34	8.5%
	21 to 30 years	58	14.5%
	31 to 40 years	152	38.0%
	40 to 50 years	102	25.5%
	Above 50 years	54	13.5%
	Total	400	100.0%
Education level	High school, Diploma, Pre-degrees	42	10.5%
	Bachelor’s degree	196	49.0%
	Master or Post Graduation	92	23.0%
	Doctor of Philosophy	70	17.5%
	Total	400	100.0%
Profession	Public sector (Government)	154	38.5%
	Private sector	246	61.5%
	Total	400	100.0%
Position	Team member	210	52.5%
	Manager or Head of the department	70	17.5%
	Director/General Manager	120	30.0%
	Total	400	100.0%
Gamification awareness	Yes	328	82.0%
	No	72	18.0%
	Total	400	100.0%
Game at work	Yes	296	74.0%
	No	104	26.0%
	Total	400	100.0%
How often do you play the games in the organisation?	Every day	38	9.5%
	Once a week	58	14.5%
	Once a month	150	37.5%
	I rarely apply game	154	38.5%
	Total	400	100.0%

Source: Data analysis.

To find out the relationship that exists between demographic variables (Independent factors) and the factors chosen for the study namely Perceived adoption, Recognition, Usefulness, Motivation, and Productivity (Dependent factors), the Anova test was conducted. Based on the significant P values and the Partial Eta Squared, the results are summarised as follows.

Only the above given demographic variables (gender, education, and age) had a significant influence on Adoption, Recognition, and Usefulness. Most of the demographic variables did not influence the dependent variables and hence it can be inferred that the variables chosen are not significantly impacted by demographic constructs that are similar irrespective of age, gender, and qualifications.

### Descriptive statistical and reliability and validity

The model was analyzed to measure the convergent validity, discriminant validity, and reliability of the constructs. Table 2 illustrates that the loading of each constructed item has outstripped 0.7 ( Esposito et al., 2021), for each construct Cronbach’s alpha was found to be above 0.7 and the aggregate reliability is more than the standard of 0.7, signifying acceptable internal consistency and reliability of the items. Moreover, the average variance extracted (AVE) from each construct is higher than 0.5, indicating an acceptable convergent validity of the manuscript measurement model. The mean and standard deviation values of each item indicate that data are clustered around the mean.

**Table 2.** Partial Eta Squared values based on ANOVA.

Dependent variable	Independent variable	P-value	Partial Eta Squared	Impact
Perceived adoption	Age	0.000	0.087	Low
Perceived recognition	Education level	0.028	0.039	Low
Perceived usefulness	Age	0.021	0.049	Low

The model was analyzed to measure the convergent validity, discriminant validity, and reliability of the constructs. Table 3 illustrates that the loading of each constructed item has outstripped 0.7 ( Esposito et al., 2021), for each construct Cronbach’s alpha was found to be above 0.7 and the aggregate reliability is more than the standard of 0.7, signifying acceptable internal consistency and reliability of the items. Moreover, the average variance extracted (AVE) from each construct is higher than 0.5, indicating an acceptable convergent validity of the manuscript measurement model. The mean and standard deviation values of each item indicate data are clustered around the mean.

**Table 3.** Descriptive statistical and reliability and validity.

		Items	Loading	AVE	CR	Alpha	Mean	SD
PA3	<---	PA	0.736	0.745	0.762	0.749	3.61	0.883
PA2	<---	PA	0.777				3.78	0.925
PA1	<---	PA	0.705				3.28	1.194
PA4	<---	PA	0.777				3.54	0.949
PR3	<---	PR	0.842	0.741	0.780	0.789	3.39	0.964
PR2	<---	PR	0.799				3.23	1.058
PR1	<---	PR	0.748				3.62	0.958
PU3	<---	PU	0.816	0.788	0.868	0.863	3.26	1.036
PU2	<---	PU	0.771				3.29	1.039
PU1	<---	PU	0.809				3.35	0.905
PU4	<---	PU	0.755				3.16	1.097
AM3	<---	AM	0.835	0.810	0.884	0.881	3.34	1.190
AM2	<---	AM	0.812				3.63	1.126
AM1	<---	AM	0.731				3.77	1.000
AM4	<---	AM	0.858				3.23	1.126
P2	<---	P	0.842	0.832	0.940	0.938	3.69	1.008
P3	<---	P	0.886				3.64	0.986
P4	<---	P	0.834				3.35	1.081
P1	<---	P	0.859				3.64	0.956
P5	<---	P	0.784				3.53	1.154
P6	<---	P	0.864				3.55	1.054
P7	<---	P	0.743				3.75	0.901
JE2	<---	JE	0.744	0.773	0.779	0.921	3.75	0.933
JE3	<---	JE	0.859				3.59	0.972
JE4	<---	JE	0.714				3.86	0.952
JE1	<---	JE	0.771				3.79	0.825
JE5	<---	JE	0.803				3.88	0.974
JE6	<---	JE	0.78				3.68	0.950
JE7	<---	JE	0.8				3.74	0.989
JE8	<---	JE	0.7				3.67	0.891

Note: PA: Perceived Adoption; PU: Perceived Recognition; PU: Perceived Usefulness; AM: Amotivation; P: Productivity; JE: Job Engagement.

### Structural equation model for the model developed

**Figure 2.**
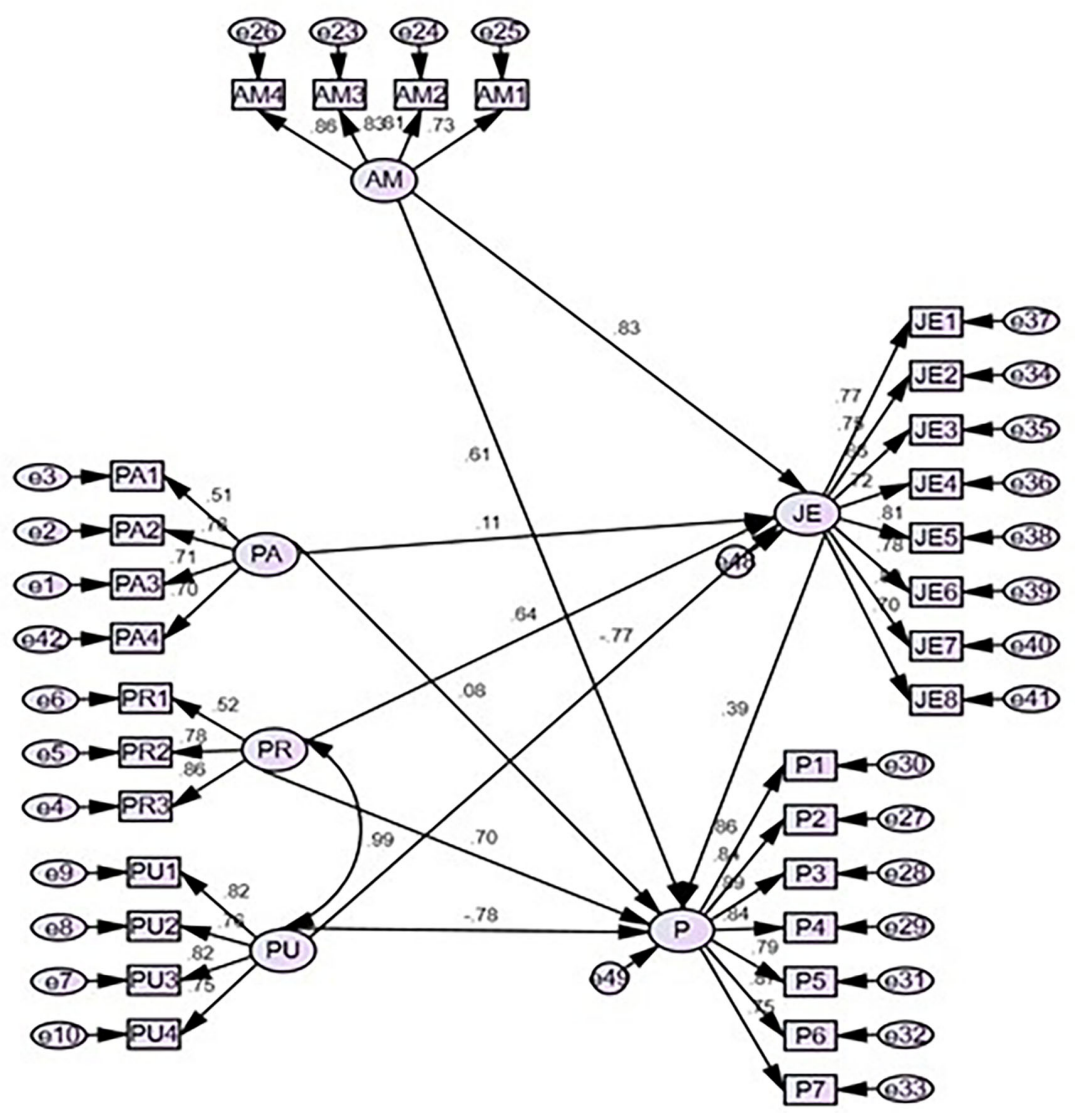
Structural equation model. Source: Data analysis.

Amos-SEM was administered in the study to understand gamification’s influence on productivity and job engagement. Figure 2 illustrates the path coefficient for the research model and all the coefficients relate to the gamified model were significant. The recommended p values for average path co-efficient and average-square must be significant at 0.05 level ( Cameron, 2013). Results indicate adequate model fit since the p-value for both these is lower than 0.05 (0.01 and 0.00) respectively. The association between perceived adoption and job engagement (H1) (β=0.017; p=0.005), perceived recognition and productivity (H2) (β=0.639; p=0.000), perceived usefulness, and job engagement (H3) (β=-0.782; p=0.000) and perceived motivation and job engagement (H4) (β=0.834; p=0.000) found to be a significant association and all four hypotheses accepted. On the other hand, mediating variables are vital factors to enhance productivity and results show in perceived adoption (H5a), job engagement mediates to enhance productivity, in contrast, is perceived recognition (H5b), perceived usefulness (H5c), and perceived motivation (H5d) job engagement does not mediate to enhance productivity. Table 4 summarizes the results of the hypothesis.

**Table 4.** Summary of hypotheses tested.

	Statement	Status	P-value
H1	Employees perceived adoption of the gamified HRM system is significantly associated with job engagement.	Accepted	<0.001
H2	Employee recognition in the gamified HRM system has a significant impact on productivity.	Accepted	<0.001
H3	Employees perceived usefulness in the gamified system is positively associated with job engagement.	Accepted	<0.001
H4	Employees perceived motivation in the gamified system is significantly associated with productivity.	Accepted	<0.001
H5	Employee Job engagement is a mediating factor to enhance productivity.	Accepted	<0.001

The percentage of variance covered by the calculated population covariance is known as the (Adjusted) Goodness of Fit. Comparable to R2, the recommended GFI and AGFI values are >0.95 and >0.90, respectively ( Marsh et al., 2019). A parsimony-adjusted index is the Root Mean Square Error of Approximation. Values that are nearer to 0 indicate a good fit. It should be either 0.05 or 0.08. The NFI has been updated to become the Comparative Fit Index. Unresponsive to sample size ( Althoff & Neiva, 2021). evaluates how well a target model fits in comparison to a null, or independent, model. It should be > 0.90 ( Marcoulides et al., 2020). The model exhibits that the CMIN/DF: is 4.580 and the p-value is <0.001. It indicates that the model is fit. The other parameters of the model are Goodness-of-Fit Index (GFI)=0.686, Adjusted Goodness-of-Fit Index (AGFI)=0.648, and Comparative Fit Index (CFI)=0.776. These parameters are above the threshold level. Based on the parameters of model results, the study concluded that the factors, perceived adoption, perceived recognition, perceived usefulness, and perceived motivation significantly influence the enhancement of job engagement and organizational productivity.

The major finding of the study notifies that 82% of the respondents are aware of gamification and are working in the private sector with minimum graduation as their qualification and in team personnel positions. Similarly, the demographic variables do not influence the dependent variables and are similar in terms of their influence. The employees perceived adoption of the gamified human resource management system is significantly associated with work engagement. In the outcome, employee recognition in the gamified HRM system also has a significant impact on productivity. The result further notifies that employees’ perceived usefulness in a gamified system has been significantly correlated with job engagement. The employees’ perceived motivation in the gamified system is also significantly associated with productivity. The outcome of the dependent variable also amplifies the similar outcome by revealing that employee job engagement is a mediating factor to enhance productivity. Overall, the factors, perceived adoption, perceived recognition, perceived usefulness, and perceived motivation significantly influence the enhancement of job engagement and organizational productivity. The underlying data and questionnaire are publicly available ( Rahiman *et al*., 2023a, 2023b).

## Discussion

Past literature emphasizes the efforts of industries to enhance employee engagement and productivity with innovative operational practices. In this framework, studies report gamification as a predominant theoretical framework that identifies and conceptualize significant mechanism to influence organizational performance and workforce engagement through various methodologies.

This study identified the role of gamification to enhance productivity and individual job engagement in various organizations. Although gamification gained popularity in various countries and notable organizations like Google, Apple, Microsoft, Cisco, and Samsung, in their operation, training and development, and human resource activities like recruitment and selection, comprehensive implementation and adoption of gamification in organizational supply chain system remain challenging ( Alhammad & Moreno, 2020). It would be challenging for the organizations in the Gulf region to implement gamification in organizational operational activities due to its low popularity. This challenge remains at the industry level where the Gulf region is dominated by energy and power sectors, and transportation and logistics in which gamification isn’t much entertained in operational level ( Mekler et al., 2017; Mitchell et al., 2017). Another vital justification for this challenge was that individuals who work in knowledge-intensive industries or companies have tasks that are naturally fun or creative where gamified systems are considered to have lesser scope. As per past studies, at the institutional level, it is challenging to integrate gamification in large firms due to the regulations ( Sailer et al., 2017). On the other hand, in small firms, gamification is an unwanted overhead since the outcome of post-implementation is not attractive. Our study drew a difference between government and private organizations in which respondents believe that it is exceedingly challenging to apply gamification in ministry or government organizations since they do not understand this process. Meanwhile, previous studies have revealed that at a strategic level, leadership-concerned challenging aspects had mostly to do with the perception of higher management of an organization ( Hammedi et al., 2021). Most of these studies opine that the top management must be tolerant enough to expect outcomes that usually do not happen in most organizations.

### Key findings

a) 82% of the respondents are aware of gamification and are working in the private sector with minimum graduation as their qualification and in team personnel positions.
b) The demographic variables do not influence the dependent variables and are similar in terms of their influence.
c) Employees perceived adoption in the gamified HRM system is significantly associated with job engagement.
d) Employee recognition in the gamified HRM system has a significant impact on productivity.
e) Employees perceived usefulness in the gamified system is positively associated with job engagement.
f) Employees perceived motivation in the gamified system is significantly associated with productivity.
g) Employee Job engagement is a mediating factor to enhance productivity.
h) The factors, perceived adoption, perceived recognition, perceived usefulness, and perceived motivation significantly influence the enhancement of job engagement and organizational productivity.

Gamification is a popular concept in the current new trends in HR. based on empirical evidence it has been proven that gamification contributes to increasing in productivity and job engagement. This research paper has shown that gamification has an impact on work engagement and productivity through pursued adoption, recognition, usefulness, and Amotivation in the organization system. Depending on the size, type, and nature of the organization the impact of gamification varies.

### Theoretical implications

The findings have significant implications for insight into how employees in the service sector are aware of the gamified working environment and react to the system through work engagement and productivity. These findings could be beneficial to industrial practitioners particularly human resources to consider certain key aspects while adopting gamified working practices. Gamification is an emerging trend in the field of human resources. Most organizations are opting for the implementation of gamification with the intention to enhance employee engagement. From application to enhance employee wellness to performance tracking gamification has been applied in every arena of HR. This research article substantiates that gamification has an impact on work engagement and productivity. However, to be effective implementation top management support and cooperation are essential. The implication of this system also differs in terms of the size and needs and resources of the organisation.

It is essential to recognize that managers should recognise the requirements of the workforce and needs to devote time to know which form of engagement procedures would be appropriate and most excellent for employee productivity. A suitable need-based execution strategy should be constructed by the organisation and pretesting of gamified sections must be performed on smaller events to commence with feasibility and effectiveness to enhance engagement and performance. These requirements and plans to change generation-wise and industry-wise, as already considered in the earlier parts of this manuscript. Finally, as gamification approaches vary among companies, personnel, and their employment profile, it is important for companies to put these elements on a canvas to assist the organizations in vigorously choosing strategies to apply the proper game mechanics. This will improve and benefit them accomplish sustainability by applying gamification for employee productivity and job engagement.

## Conclusion

People are coded genetically, just like computers and various permutations and combinations play an important role in improvising their productivity in terms of work. Gamification no doubt has been a recently sought-out tool to improve the job engagement of employees that further enhances productivity, but nevertheless, it is not the only relevant tool. In an IT-enabled work environment, gamification has shown a positive impact, but in the case of pure services like academics, and hospitals it is yet to make a mark. In order to check its relevance, the psychological perspective of individuals has to be tapped on. The study shows that there are people who have performed well though they have not used gamification. Newer methodologies or technological innovations may be needed to use gamification for different industries. That necessitates a new breed of gamification technologies that are tailor-made to suit the needs of different classes of employees. Further research may be warranted in this perspective of widening the scope of gamification.

### Limitations & future research

The research limitations should be taken into account when interpreting the results. The research was conducted *via* an electronic survey, which is susceptible to frequent method bias. Data were only collected in Gulf countries only, which limits the generalizability of the findings. The study was based on a sample survey and was not experimental, hence it does not fall under the gamut of causality. Further, the empirical study is based upon only those institutions and organizations which included gamification as a part of their work. Respondents who were working in an organization without gamification would have increased their knowledge with regard to the impact of gamification on productivity.

In conclusion, it can be written that gamification is quite a new terminology and hence it warrants full-fledged research in this domain. This research paper is an attempt to showcase the usage of gamification in a single sector and can prove to be a vital point for further research.

## Authors’ contributions

**Habeeb Ur Rahiman:** *Conceptualization, Formal Analysis, Funding Acquisition, Investigation, Methodology, Resources, Supervision, Writing – Original Draft Preparation*

**Rashmi Kodikal:** *Conceptualization, Data Curation, Formal Analysis, Investigation, Methodology, Supervision, Validation, Writing – Original Draft Preparation, Writing – Review & Editing*

**Dr. Sucharitha Suresh:** *Software, Validation, Visualization, Writing – Review & Editing*

## Data Availability

Figshare: Game on: Can gamification enhance productivity?, https://doi.org/10.6084/m9.figshare.22083158 ( Rahiman *et al*., 2023a). The project contains the following underlying data:
- Gamification and Productivity Data.sav (Questionnaire responses) Figshare: Questionnaire. https://doi.org/10.6084/m9.figshare.22262974 ( Rahiman *et al*., 2023b). The project contains the following extended data:
- Questionnair Gamification.pdf Data are available under the terms of the Creative Commons Zero “No rights reserved” data waiver (CC0 1.0 Public domain dedication).
