# Supplementary material for: Game on: Can gamification enhance productivity?
Source: F1000Res. 2023 Oct 18;12:818. Originally published 2023 Jul 12. [Version 2] doi: 10.12688/f1000research.131579.2 (PMC10905147; doi:10.12688/f1000research.131579.2)
Supplement: Peer review history

# Reviewer report: Reviewer response for version 2

**DOI:** 10.5256/f1000research.157243.r225218

**Published:** 2024-02-21

**Authors:** John Aliu

**Affiliations:** 1 Institute for Resilient Infrastructure Systems, University of Georgia, Athens, Georgia, USA

**Introduction:**

1. In the second sentence of introduction section, it says….”In an era of high-speed digitalized technology and a multi-generational capacity team, the scenario has resulted in a struggle for the smarted of the people”… That should be ‘smartest’ Grammar should be amended.
2. Use active voice where possible to enhance the directness and clarity of your statements.
3. Also, maintain consistency in verb tense throughout the introduction. For example, if discussing past research findings, use the past tense consistently.

**Literature review:**

1. Define acronyms upon first use to facilitate understanding, for example, AI, ML, and IoT.
2. Expand on the explanation of how gamification is applied in HRM systems. Provide examples or case studies to illustrate its practical implementation and impact

**Research Methodology:**

1. Explain the rationale behind choosing the Gulf Cooperation Council (GCC) countries for the study. Discuss why the selected sectors (IT, banking, education, and telecom) were deemed relevant to the research objectives.
2. Clarify the steps involved in the multistage sampling method. Provide details on how companies were selected within each sector and how judgment-based sampling was applied.

Is the work clearly and accurately presented and does it cite the current literature?

Yes

If applicable, is the statistical analysis and its interpretation appropriate?

Yes

Are all the source data underlying the results available to ensure full reproducibility?

Yes

Is the study design appropriate and is the work technically sound?

Yes

Are the conclusions drawn adequately supported by the results?

Yes

Are sufficient details of methods and analysis provided to allow replication by others?

Yes

Reviewer Expertise:

Sustainable construction, Construction Digitalization

I confirm that I have read this submission and believe that I have an appropriate level of expertise to confirm that it is of an acceptable scientific standard, however I have significant reservations, as outlined above.

# Reviewer report: Reviewer response for version 2

**DOI:** 10.5256/f1000research.157243.r225213

**Published:** 2023-12-18

**Authors:** Martin Kiselicki

**Affiliations:** 1 Department of Economics, Ss Cyril and Methodius University, Skopje, North Macedonia

The article is focused on researching the usefulness of gamification implementation in various industries in the GCC region. The authors did diligent work on the introductory and literature review sections of the paper, clearly demonstrating the power of gamification and various scientific research focused on the model. The most important elements, mechanisms and dynamics of gamification are clearly outlined. 

 Regarding the primary research, authors are focusing on a survey questionnaire and statistical analysis of the data. The proposed methodology is consistent with previous research on the topics, as well as the SEM model and related hypothesis. I would only point out that the research has been done in other regions (as well as on a global scale), so there is only partial contributions to further the field. 

 Since the methodology section is transparently explained, other authors can contribute by utilizing the same research design in similar research. Results are in line with the raw data, as well as the relevant statistical metrics and levels of relevance.

Is the work clearly and accurately presented and does it cite the current literature?

Yes

If applicable, is the statistical analysis and its interpretation appropriate?

Partly

Are all the source data underlying the results available to ensure full reproducibility?

Yes

Is the study design appropriate and is the work technically sound?

Partly

Are the conclusions drawn adequately supported by the results?

Yes

Are sufficient details of methods and analysis provided to allow replication by others?

Yes

Reviewer Expertise:

Organizational sciences, Gamification in education

I confirm that I have read this submission and believe that I have an appropriate level of expertise to confirm that it is of an acceptable scientific standard.

# Reviewer report: Reviewer response for version 1

**DOI:** 10.5256/f1000research.144432.r189162

**Published:** 2023-09-08

**Authors:** Ola Olayinka

**Affiliations:** 1 Computer Science, The University of Sheffield, Sheffield, England, UK

Interesting paper covering gamification and its application in organisations in the gulf region. The paper was mostly well written and clear to read with some recent papers in the field cited.

 However, there are a number of issues that need to be clarified or better presented.

1. ".. our research was taken up to examine the impact of gamification in various organizations and simultaneously see its relationship with job engagement and productivity. The primary objective of this research is to identify the role of perceived usefulness, motivation, adoption, and recognition in the organization while adopting gamified tasks in an HR system." It is not entirely clear what the focus of the paper is, examining impact of gamification in organizations, examining its relationship with engagement and productivity or identifying the role of perceived usefulness, motivation, adoption, and recognition in when gamification is used, or a combination of three of them.
  From the hypothesis, it is clear that you are trying to understand how perceived usefulness and perceived adoption of gamification in HRM systems influences engagement and motivation. I will suggest that it is made clear in the above quote.
2. I will suggest general proof-reading of the article as there are some grammatical errors that can make reading the article a bit difficult to understand.
3. I will suggest the inclusion of a detailed literature review section that critically assesses and discusses other similar research.
4. A bit more needs to be done in the methodology section. There needs to be more information provided about the research sample and its appropriateness.  How many respondents were from each country? The lack of clarity allows for the generalisability of the results to be questioned even in the gulf region.
5. There are a number of sentences (or claims) in the discussion section that you need to justify with appropriate references.
  e.g. "In this framework, studies report gamification as a predominant theoretical framework that identifies and conceptualizes a significant mechanism to influence organisational performance and workforce engagement through various methodologies." What studies are these?
  "It would be challenging for the organisations in the Gulf region to implement gamification in organisational operational activities due to its low popularity. " Please provide a reference.  
  "On the other hand, in small firms, gamification is an unwanted overhead since the outcome of post-implementation is not attractive. ". Please provide a reference.
6. Some parts of the discussion were not clear. e.g. "Our study drew a difference between government and private organizations in which respondents believe that it is exceedingly challenging to apply gamification in ministry or government organizations since they do not understand this process." How was this done? It was not clear from the methodology or objectives that this was a goal.
7. There needs to be more discussed about the findings in the discussion section. This section seems quite weak and omits key findings that the reader would like to know.

Is the work clearly and accurately presented and does it cite the current literature?

Partly

If applicable, is the statistical analysis and its interpretation appropriate?

Partly

Are all the source data underlying the results available to ensure full reproducibility?

Partly

Is the study design appropriate and is the work technically sound?

Partly

Are the conclusions drawn adequately supported by the results?

No

Are sufficient details of methods and analysis provided to allow replication by others?

No

Reviewer Expertise:

Gamification,  information systems, research methods and qualitative research.

I confirm that I have read this submission and believe that I have an appropriate level of expertise to state that I do not consider it to be of an acceptable scientific standard, for reasons outlined above.

# Response

**Published:** 2023-10-08

**Authors:** Habeeb Ur Rahiman

**Affiliations:** Kingdom University, Bahrain

Dear Reviewer,

 We would like to express our deep gratitude for your invaluable feedback and the dedicated time you invested in reviewing our research. Your insightful comments have played a pivotal role in guiding our efforts to enhance the overall quality and rigor of our study. Below, we provide comprehensive responses to each of the points you raised.

 Section Enhancement: We have diligently implemented the changes recommended for the specified section, ensuring that your valuable suggestions have been incorporated effectively.

 Proofreading and Quality Assurance: In response to your suggestions, we conducted a thorough proofreading of our work, addressing any grammatical and language-related concerns to ensure the utmost clarity and readability.

 Literature review: While the journal requested a title labeled as "Background," we have adjusted it to align with your preference, now designating it as "Literature Review" to accurately represent its content and purpose.

 Methodology Refinement: Following your guidance, we have made the necessary revisions in the methodology section. Specifically, we have provided a detailed breakdown of the sample respondents, highlighting the distribution by country for enhanced clarity and transparency.

 Discussion Restructuring: We have undertaken a comprehensive restructuring of the discussion section, addressing instances where references were missing and ensuring that all relevant points are now properly cited. Additionally, we have enriched the section by incorporating a more extensive set of references to further elucidate our results.

 We sincerely hope that these adjustments meet your expectations and align with the standards expected for publication. If you find the need for any further modifications or have additional suggestions, please do not hesitate to communicate them to us. Your continued guidance is highly valued and greatly appreciated.

 Warm regards,

# Reviewer report: Reviewer response for version 1

**DOI:** 10.5256/f1000research.144432.r186391

**Published:** 2023-07-31

**Authors:** Easwaramoorthy Rangaswamy

**Affiliations:** 1 Amity Global Institute, Singapore, Singapore

The manuscript focuses on one of the contemporary issues, which I think is very interesting and a complex issue too. The authors have managed to articulate the issue reasonably well in the introduction.

 The introduction part can have a subsection about the research problem and can also show the research questions clearly.

 The conceptual model can be aligned with the Literature review and the proposed hypotheses. Also, ensure that the latest literature is provided. There are not many 2022/2023 reviews provided.

 Methodologically, the paper does not lack rigour, but it needs more clarity on the sample selected, demographics and also potential limitations due to the methodology. It does not mention the “representativeness of the sample”. The methodology section need also to include details about the research design, research philosophy, research approach, etc. Provide a clear and detailed explanation of the research design and methodology employed in the study. Clearly state the research approach and justify its suitability for addressing the research questions.

 The section “Ethical Considerations” mentioned, “The data collected will be used only for academic research purposes”. It is suggested to write in the past tense, as the data collected is not in the report stage.

 The results and findings section should go beyond a mere summary and interpretation of the findings. Provide an in-depth analysis of the data, relating it back to the research questions and objectives. Clearly explain the implications of the findings and their significance in the context of the prevailing literature available. Additionally, discuss any unexpected or contradictory findings and offer potential explanations. I see that certain findings are discussed but I also see a sub-section named “key findings” in section Discussion. Looks duplicated. Also, avoid point-wise writing in “key findings”.

 The discussion section should include a comprehensive review and synthesis of relevant literature. Compare the findings of the study with existing research in the research area. Identify areas of agreement or divergence and discuss the potential reasons behind these differences. This will strengthen the validity and generalizability of the study's findings.

 Review the manuscript for coherence and flow, ensuring the content is well-organized and logically structured.

Is the work clearly and accurately presented and does it cite the current literature?

Partly

If applicable, is the statistical analysis and its interpretation appropriate?

Partly

Are all the source data underlying the results available to ensure full reproducibility?

Partly

Is the study design appropriate and is the work technically sound?

Partly

Are the conclusions drawn adequately supported by the results?

Partly

Are sufficient details of methods and analysis provided to allow replication by others?

Partly

Reviewer Expertise:

Management

I confirm that I have read this submission and believe that I have an appropriate level of expertise to confirm that it is of an acceptable scientific standard, however I have significant reservations, as outlined above.
